# Gene Expression Changes in the Colon Epithelium Are Similar to Those of Intact Colon during Late Inflammation in Interleukin-10 Gene Deficient Mice

**DOI:** 10.1371/journal.pone.0063251

**Published:** 2013-05-20

**Authors:** Anna E. Russ, Jason S. Peters, Warren C. McNabb, Matthew P. G. Barnett, Rachel C. Anderson, Zaneta Park, Shuotun Zhu, Paul Maclean, Wayne Young, Gordon W. Reynolds, Nicole C. Roy

**Affiliations:** 1 Food Nutrition & Health Team, Food & Bio-based Products Group, AgResearch Grasslands, Palmerston North, New Zealand; 2 Institute of Food Nutrition and Human Health, Massey University, Palmerston North, New Zealand; 3 Riddet Institute, Massey University, Palmerston North, New Zealand; 4 AgResearch Grasslands, Palmerston North, New Zealand; 5 Bioinformatics & Statistics, AgResearch, Hamilton, New Zealand; 6 Discipline of Nutrition, Faculty of Medicine and Health Sciences, The University of Auckland, Auckland, New Zealand; 7 Auckland Cancer Society Research Centre, The University of Auckland, Auckland, New Zealand; University of Jaén, Spain

## Abstract

In addition to their role in absorption and secretion, epithelial cells play an important role in the protection of the colon mucosa from the resident microbiota and are important for the maintenance of homeostasis. Microarray analysis of intact colon samples is widely used to gain an overview of the cellular pathways and processes that are active in the colon during inflammation. Laser microdissection of colon epithelial cells allows a more targeted analysis of molecular pathways in the mucosa, preceding and during inflammation, with potentially increased sensitivity to changes in specific cell populations. The aim of this study was to investigate the molecular changes that occur in early and late inflammation stages in colon epithelium of a mouse model of inflammatory bowel diseases. Microarray analysis of intact colon samples and microdissected colon epithelial cell samples from interleukin-10 gene deficient and control mice at 6 and 12 weeks of age was undertaken. Results of gene set enrichment analysis showed that more immune-related pathways were identified between interleukin-10 gene deficient and control mice at 6 weeks of age in epithelial cells than intact colon. This suggests that targeting epithelial cells could increase sensitivity for detecting immune changes that occur early in the inflammatory process. However, in the later stages of inflammation, microarray analyses of intact colon and epithelium both provide a similar overview of gene expression changes in the colon mucosa at the pathway level.

## Introduction

Laser microdissection (LMD) is a technique that allows pure populations of cells to be removed from frozen tissue, enabling subsequent molecular profiling of these cells which is then reflective of their *in vivo* state [1], [2], [3]. An example of how LMD can contribute to understanding the development of a disease is a study of the development of *Helicobacter*-induced gastric lymphomas [4]. Here, the authors demonstrated that gene expression patterns in lymphocytic and mucosal fractions differed from each other, and from expression patterns in whole stomach. These findings confirm that the cellular origin of particular transcripts could be identified by analysis of gene expression in specific cell fractions.

Gene expression analysis in the large intestine has been performed in a number of studies using mouse models of inflammatory bowel disease (IBD) and biopsy tissues of IBD patients [5], [6], [7]. Such analyses have mostly been performed on intact colon tissue and provide insights as to the molecular pathways involved in the initiation and maintenance of inflammation. However, the colon is a complex tissue with distinct layers (mucosa, submucosa, muscular layers and serosa) and numerous cell types in each layer. The epithelial layer of the colon mucosa contains enterocytes, goblet cells, stem cells and intraepithelial lymphocytes. The cellular location of gene expression signals cannot be determined in intact colon sections, and expression signals in lower-abundance cell populations may be missed due to noise from gene expression changes in more abundant cell types. The ability to analyse gene expression profiles in specific cell types or tissue regions using LMD may provide a more detailed understanding of the function of the various cells and types of tissue within the colon preceding and during inflammation. As yet, there have been no reports of global gene expression profiles in colon epithelium before and after the development of colitis. Furthermore, while analysis of gene expression in inflamed colon epithelium tissues has been undertaken in our laboratory [8], where the epithelial layer of the colon was obtained by manual scraping, LMD represents a novel and more accurate method of isolating epithelial cells from colon tissue than previously reported in this context.

Intact inflamed colon includes a large number of infiltrating immune cells, which may be associated with tissue damage and the gross changes in intestinal morphology that occur in inflammation. However, these immune cells may be less involved with the initiation and maintenance of the pro-inflammatory signals that underlie inflammation than endogenous colon mucosal cells. Changes in the colonic epithelium, such as increased paracellular permeability and mucosal immune defence malfunction, are thought to be important in the initiation of inflammation [9], [10] and its response to commensal bacteria [11], [12]. Disruption of the epithelial barrier allows entry of bacterial antigen into the mucosa which may lead to an uncontrolled immune response to the luminal microbiota and inappropriate inflammatory response. Studies of gene expression changes in the epithelium under inflammatory conditions often use cultured epithelial cells, which may not be representative of the *in vivo* state of the epithelium in IBD or animal models of colitis. A previous study involving microarray analysis of microdissected epithelial cells in colitis only reported expression changes in a small number of genes [13]. The central role of the epithelium in inflammation development requires a further understanding of the cellular changes occurring in the epithelial layer in IBD *in vivo*.

The hypothesis of this study was that LMD and subsequent microarray analysis of colon epithelial cells would enable the identification of pathways and processes related to the inflammatory process that are not detected by microarray analysis of intact colon. To investigate this hypothesis, we used the interleukin 10 gene-deficient (*Il10^−/−^*) mouse, which develops intestinal inflammation similar to that seen in IBD, and has therefore been well characterized and extensively employed as an animal model of human IBD [5], [14], [15]. The first aim was to characterize global gene expression changes in intact colon tissue before and after inflammation developed in *Il10^−/−^* mice (at 6 and 12 weeks of age, respectively). This was intended to better understand the molecular changes occurring in the colon in the early stage of inflammation, before any measurable changes in morphology occur. Whole colon profiles were assessed at 12 weeks of age to confirm that the gene expression changes occurring in this model are similar to other gene expression profiles in IBD and previous experiments using this model in our laboratory [7], [14], [16]. The second aim was to examine whether the global gene expression profile of colon epithelial cells is similar to that of whole colon tissue and describe any differences between the profiles to determine whether intact colon provides a good approximation of the changes in the epithelium in early and late inflammation. Findings indicated that LMD followed by microarray profiling of gene expression identified additional immune-related pathways at 6 weeks of age, in agreement with the hypothesis, but in contrast, profiles at 12 weeks of age were similar between intact colon and epithelium.

## Methods

### Animals

#### Ethics statement

This study was carried out in strict accordance with the recommendations of the New Zealand Animal Welfare Act 1999. The protocol was approved by the AgResearch Limited (Ruakura) Animal Ethics Committee (Ethics Approval No.: 11343). All efforts were made to minimize suffering.

Nineteen *Il10^−/−^* mice (C57BL/6J background; B6.129P2-*Il10^tm1Cgn^*/J), aged 5–6 weeks, were purchased from the Jackson Laboratory (Maine, USA) and 16 control C57BL/6JArc mice, also 5–6 weeks of age, were obtained from the Small Animal Colony (AgResearch Ruakura, Hamilton, New Zealand). Mice were inoculated with a mixture of *Enterococcus* species and conventional intestinal microbiota at approximately 6 weeks of age in order to produce reliable and consistent levels of colon inflammation by 12 weeks of age using the method previously reported [14].

Mice were housed in conventional conditions, in a room with 12 hour light/dark cycle, and controlled temperature (22±1°C) and humidity (approximately 50%). Mice were contained in shoebox-style polycarbonate cages with wire lids, lined with recycled paper litter with tissue for nest building and a plastic tube or hut for environmental enrichment. Mice were provided 10 g of non-sterile powdered AIN76A diet, made in-house [17], [18], [19], which was sufficient to provide *ad libitum* intake throughout the study. Refusals were measured 3–4 times weekly. Water was provided *ad libitum* (refreshed weekly) and cages were cleaned and autoclaved weekly. Mice were checked every day for general appearance and behaviour, and disease symptoms (such as weight loss, diarrhoea, and inactivity) using the General Health Score (GHS) [20], and weighed 3–4 times weekly. Mice which reached a GHS of 3 (a score of 1 was considered healthy while 5 was near death) were promptly euthanized.

Mice were euthanized at 5–6 weeks for the early inflammation time-point (three days post-inoculation, with no inflammation detected by histopathology) or 11–12 weeks for the maximal-inflammation time-point [16]. Immediately prior to sampling, mice were fasted for 14 hours overnight, re-fed for 2 hours and fasted again for 2 hours in order to minimize variation in food intake and gene expression due to differences in intake [21]. Mice were weighed just prior to euthanasia via CO_2_ asphyxiation and cervical dislocation. The intestine was carefully removed and cut lengthwise while being rolled around a plastic 10 mL pipette, then flushed with cold, sterile 0.9% NaCl to remove digesta, laid out on an ice cold stainless steel tray and sectioned. Sections of duodenum, jejunum, ileum, and colon were fixed in 10% phosphate buffered formalin for histology and stored at room temperature.

The proximal colon was sectioned into three pieces. Approximately one centimetre of the proximal end was kept intact rather than being cut lengthwise, which was then further cut into two halves. The most proximal half was embedded in Optimal Cutting Temperature medium (Tissue-Tek OCT, Sakura Finetek U.S.A., Inc., Torrance, California, USA) and stored at −80°C for gene expression analysis of laser microdissected cells. Colon epithelial cells were isolated from this tissue for microarray analysis of gene expression. The other half of the uncut proximal colon section was preserved for histopathology as described above. From the remaining colon, which was cut lengthwise, the most proximal half centimetre (approximately 20 mg) was preserved in 1 mL of RNAlater (Ambion Inc., Austin, Texas, USA) to improve preservation of RNA integrity, stored at 4°C overnight, and snap-frozen the next morning after removal of excess liquid for long-term storage at −80°C. This was used for gene expression analysis of the “intact colon”, a representative section of full-thickness proximal colon.

### Measurement of Intestinal Inflammation

The intestine of each mouse was photographed before sectioning as a secondary measure of intestinal inflammation. The gastrointestinal tract (GIT) was unrolled from the pipette onto an ice-cold stainless steel tray with attached ruler, to enable subsequent estimation of the intestinal area using ImageJ software [22].

Inflammation in all intestinal regions was determined by histological examination as previously described. Briefly, formalin-fixed samples were processed, sectioned, stained with haematoxylin and eosin and evaluated for inflammation under a light microscope, using a modification of a previously described scoring system [23], [24]. A histological injury score (HIS) was assigned based on the presence of inflammatory lesions, tissue destruction and tissue repair [25].

### Statistical Analysis of Weight, Intake and Intestinal Area and Histology Scores

Initial and final weight and intake data, and intestinal area, were analysed using an Analysis of Variance (ANOVA). Differences between means were considered significant when the probability of this difference occurring purely by chance was less than 0.05. An unbalanced ANOVA was used to analyse the histology scores with scores (log +0.05)-transformed before analysis due to the large number of zero values in the data. All statistical analyses were performed using GenStat edition 11 (VSN International Ltd, Hemel Hempstead, Hertfordshire, UK).

### Microdissection of Epithelial Cells

Colon samples were embedded using a methodology that ensured that the colon was maintained in its natural shape in cross-section resulting in excellent visualisation on the laser microdissector. Immediately on removing the intestinal tissue from the animal, the tissue was rinsed with saline, trimmed, any fat removed, and embedded on a stainless steel block placed on dry ice (3–4 colon lengths per block), with the cut end in a downward orientation so that it was supported in an optimal position for cryosectioning. The embedding material was poured over and allowed to slowly freeze. Samples were stored at −85°C prior to cryosectioning, which was undertaken using a Leica CM1950 cryostat (Leica Microsystems Gmbh, Wetzler, Germany), and 8 µm thick sections were then placed on an RNase-free metal-framed membrane slide (Molecular Machines and Industries AG, Zurich, Switzerland).

**Figure 1:**
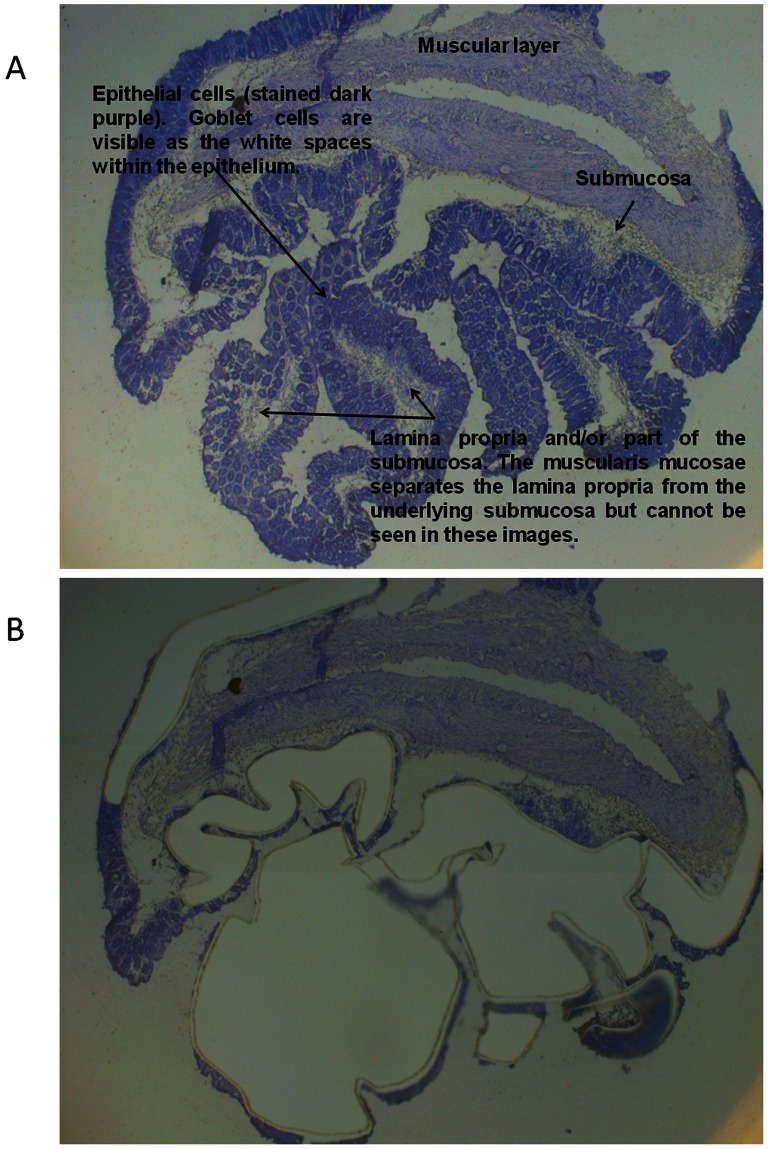
Non-inflamed mouse colon. Colon section for mouse 18, an *Il10^−/−^* mouse sampled at 6 weeks of age, before microdissection of epithelial cells (A) and after microdissection of epithelial cells (B). The lamina propria was taken from this section along with the epithelial cells but is mostly connective tissue in the non-inflamed mice.

**Figure 2:**
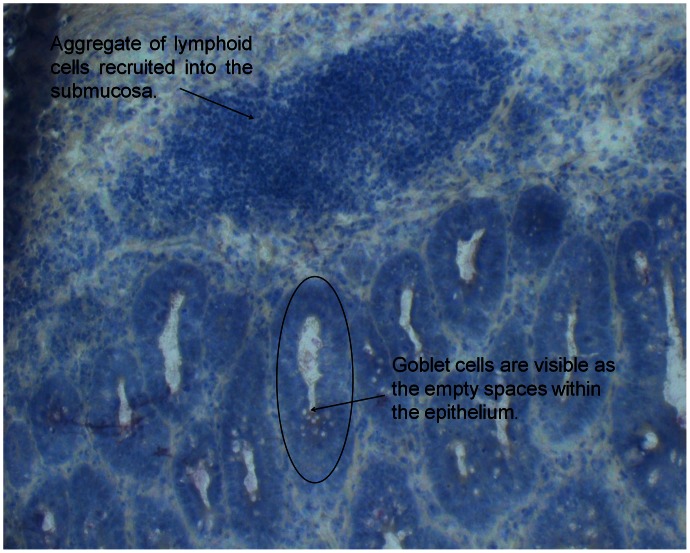
Inflamed mouse colon. Colon section from mouse 17, an *Il10^−/−^* mouse sampled at 12 weeks of age, before microdissection of epithelial cells. The aggregate of immune cells is visible in the mucosa, which is typical for CD-like colitis, such as that seen in this mouse model. The circle outlines epithelial cells in a colonic crypt, which were cut out for analysis of gene expression.

Staining was performed using the HistoGene LCM Frozen Section Staining Kit (Arcturus Bioscience Inc., Mountain View, California, USA) according to the manufacturer’s instructions with minor modifications: removal of the post-stain water step and removal of the final xylene step [26]. All containers housing stain solutions were kept in an aluminium cold block to minimize the activity of any contaminating RNases. All staining equipment was treated with UV light prior to use to destroy any contaminating nucleic acids or proteins. Slides were stored in a dessicator until LMD was performed to prevent rehydration of the cells and reactivation of endogenous RNases. LMD of epithelial cells was performed using the mmi CellCut laser microdissector (Molecular Machines and Industries AG, Zurich, Switzerland) within 45 minutes of the final staining step. Examples of the cells that were dissected out for the epithelial cell microarray analysis from non-inflamed and inflamed mice are depicted in Figure 1 and Figure 2, respectively. Lamina propria was excluded from the sample as much as possible in order to obtain epithelial cells only.

### Microarray Analysis of Gene Expression

A reference design was used for each microarray experiment, which involved hybridising an experimental sample (representing a single animal) labelled with Cy3 dye and a reference RNA sample labelled with Cy5 dye to each microarray. The design was generated using CycDesigN 4.0 (CycSoftware Ltd, Ranfurly, NZ). Six mice per treatment were selected for microarray analysis such that the full range of colon HIS in each treatment group was represented. When mice within a group had similar colon HIS scores, representative animals were randomly selected for microarray. Treatments were randomized and evenly spread across microarray slides so that array or slide effects could be accounted for in the analysis.

Total RNA was extracted from up to 20 mg sections of intact (not microdissected) proximal colon using Trizol (Invitrogen, Carlsbad, California, USA), followed by purification of the RNA using RNeasy columns (Qiagen Inc., Valencia, California, USA). The purity and yield of RNA was assessed using the NanoDrop 1000 (Thermo Scientific, Wilmington, Delaware, USA). RNA Integrity Number (RIN) was measured, and concentration estimated, for each sample using the RNA 6000 Nano Chip Kit for the 2100 Bioanalyzer (Agilent Technologies, Santa Clara, California, USA). Samples were only used in subsequent analyses if they were of high purity (absorbance ratios A260/A280 greater than 1.8 and A260/A230 greater than 1.6) and integrity (RIN greater than 8.0). Where there was evidence of impurities such as proteins, chaotropic salts or phenol being present (as indicated by a low A260/A230 ratio), the samples were further purified using ammonium acetate precipitation with glycogen (Ambion Inc., Austin, Texas, USA) as a carrier (according to the manufacturer’s instructions) to remove these impurities and thus increase the purity of the precipitated RNA.

RNA was extracted from the microdissected colon epithelial cells from sections of proximal colon using the PicoPure RNA Isolation Kit (Arcturus Bioscience Inc., Mountain View, California, USA) following the manufacturer’s instructions (User Guide Version D) and included the recommended DNase treatment using the RNase-free DNase Set (Qiagen Inc., Valencia, California, USA). The integrity of the extracted total RNA was verified using the RNA 6000 Pico Chip Kit for the 2100 Bioanalyzer. Only samples with two strong ribosomal RNA peaks or a RIN of at least 4.6 were used for microarray (the range of RINs obtained for the samples used in the microarray experiment was 4.6 – 6.9, with a mean RIN of 5.5). The Qubit 2.0 Fluorometer (Invitrogen, Carlsbad, California, USA) was used to quantify RNA samples obtained from microdissection. Where concentration of the RNA sample was too low to be measured by the Qubit (original sample <4 ng/µL), the maximum volume (10 µL) of sample was amplified for the microarray experiment. Up to 15 ng/µL of RNA in a total volume of 30 µL was obtained from the epithelium of two 8 µm thick colon sections, with most samples yielding less than 10 ng/µL.

For the intact colon samples, extracted colon RNA (500 ng) was amplified and labelled using the Low Input Linear Amplification (Two-Colour) kit. For the microdissected samples, extracted total RNA (30 ng in a maximum volume of 10 µL) was linearly amplified using the RiboAmp HS RNA Amplification Kit for two rounds of amplification (Arcturus Bioscience Inc., Mountain View, California, USA). Amplified cRNA (10 µg in a maximum volume of 40 µL) was labelled for microarray hybridisation using the Turbo Labelling kits for Cy3 and Cy5 dyes (Arcturus Bioscience Inc., Mountain View, California, USA).

The purity and fluorescent intensity of the amplified and labelled cRNA was assessed using the NanoDrop 1000 (Thermo Scientific, Wilmington, Delaware, USA). The amplification and labelling procedure was repeated if a sample did not meet the requirements of specific activity >8.0 pmol Cy3 or Cy5 per µg of cRNA and yield >825 ng. Samples were also run on the 2100 Bioanalyzer (Agilent Technologies, Santa Clara, California, USA) using the RNA 6000 Nano Chip Kit to confirm that a range of fragment lengths were present. One sample obtained via LMD did not label adequately so samples from only five C57BL/6J mice at 12 weeks of age could be included in the epithelial cell microarray experiment rather than the intended six mice per group.

Amplified cDNA from each mouse was labelled with Cy3 dye and hybridized to one mouse 44 K oligonucleotide two-colour array (Whole Mouse Genome Oligo Microarray Kit, 4×44 K; version 1) (Agilent Technologies, Santa Clara, California, USA), along with cyanine (Cy) 5-labelled reference cDNA that was prepared in one large pool from total RNA extracted from normal healthy Swiss mouse tissue (including small intestine, colon, kidney, and liver of adult mice, plus foetal tissue). Hybridisation was performed using the Gene Expression Hybridization kit (Agilent Technologies) according to the manufacturer instructions. Cy 3-CTP and Cy 5-CTP dye (10 mM) for the intact colon microarrays in the time-course experiment was obtained from Perkin-Elmer (Boston, MA, USA) whereas Cy dye was included in the Agilent amplification kits for the epithelial cell microarray analyses. Slides were scanned using a DNA Microarray Scanner G2565CA (Agilent Technologies, Santa Clara, California, USA) according to the manufacturer’s instructions. Array images (TIFF files) were uploaded into Agilent Feature Extraction Software (version 8.5.1.1 for intact colon arrays or 10.10.1.1 for epithelial arrays), which generated numerical intensity data for each probe, as well as a quality control report for each array. The data files generated by the Feature Extraction software were used for further analysis of gene expression data.

Quality control and generation of gene lists from Feature Extraction data was performed using the Bioconductor R package (R 2.12.1; R Foundation for Statistical Computing, Vienna, Austria). Background correction was not necessary for any of the microarray experiments due to homogeneous hybridisation [27]. Analysis of the raw microarray data was performed using the Linear models for microarray analysis (Limma) package for Bioconductor (http://www.bioconductor.org/). Control and bad-flagged spots were removed from the analysis. Intensity ratio values for all spots were normalized using a global loess smoothing procedure to remove the effect of systematic variation on the microarrays [28], [29].

The normalized data from the arrays of each treatment group (age and mouse strain) were averaged. For each treatment comparison, a list of differentially expressed probe sets was generated by calculating a moderated t-statistic and false discovery rate (FDR) [30] for each probe set using an empirical Bayes approach in the Limma package. Expression changes were considered significant when absolute biological fold change (FC) was greater than 1.5 and FDR was less than 0.05. The data discussed in this publication have been deposited in NCBI’s Gene Expression Omnibus [31] and are accessible through GEO Series accession number GSE39859 (http://www.ncbi.nlm.nih.gov/geo).

Ingenuity Pathway Analysis (IPA; Version 9.0, Ingenuity Systems, Inc., Redwood City, California, USA; http://www.ingenuity.com), was used to perform network and pathway analyses. The full Limma-generated gene lists for each treatment comparison were uploaded into IPA and the following cutoffs applied to select differentially expressed genes for further analysis: absolute FC greater than 1.5 and FDR less than 0.05. Canonical pathways were considered to be significant when at least 10% of the genes from a particular pathway were differentially expressed in the microarray dataset and a probability of less than 0.05 was calculated by Fisher’s exact test.

Venny software (http://bioinfogp.cnb.csic.es/tools/venny/index.html) was used to generate Venn diagrams showing the number of genes each treatment comparison had in common [32].

Gene set enrichment analysis (GSEA) was performed using the Bioconductor R package (R 2.12.1; R Foundation for Statistical Computing, Vienna, Austria) on the normalized data using the Kyoto Encyclopedia of Genes and Genomes (KEGG) pathway database. Unlike gene-centric analyses, GSEA takes into account the expression of a group of genes as a whole, rather than examining individual genes. While individual genes may not show differential expression as determined by predefined *P* value and fold change cut-offs, alterations in the activity of biological pathways may be manifested by small, but consistent changes in expression of multiple genes within that pathway. Analysis of microarray data using GSEA may detect changes in these pathways that would otherwise be overlooked by gene-centric methods. Only pathways with permutation P values less than 0.05 were considered significant.

A list of immune-related genes was generated using the gplots library and heatmap.2 command in R to create a heatmap of immune-related genes in intact colon and colon epithelium. In IPA, all genes present in immune-related networks for the comparison *Il10^−/−^* mice at 12 weeks of age versus *Il10^−/−^* mice at 6 weeks of age, in both intact colon and colon epithelium, were combined into a list. This list was further limited to only those genes that were differentially expressed by at least 2-fold in the comparisons *Il10^−/−^* mice at 12 weeks of age versus *Il10^−/−^* mice at 6 weeks of age and *Il10^−/−^* mice at 12 weeks of age versus C57BL/6J mice at 12 weeks of age in intact colon or epithelium. A measure of the similarity between treatments, the Pearson correlation coefficient, was calculated using a correlation analysis in R.

### qPCR Validation of Microarray Results

The expression of 6 genes was quantified using real-time quantification polymerase chain reaction analysis (qPCR). The genes selected for analysis were: peroxisome proliferator-activated receptor α (*Ppara*; NM_011144; PrimeTime assay ID Mm.PT.51.7020742), signal transducer and activator of transcription 1 (*Stat1*; NM_009283; Mm.PT.51.19067632), transporter 2, ATP-binding cassette, sub-family B (MDR/TAP) (*Tap2*; NM_011530; Mm.PT.51.9501990.gs), interleukin 18 (*Il18*; NM_008360; Mm.PT.51.11096643), matrix metalloproteinase 3 (*Mmp3*; NM_010809; Mm.PT.51.16575457.gs), and S100 calcium binding protein G (*S100 g*; NM_009789; Mm.PT.51.7385865).

For qPCR analysis, 500 ng of total RNA was reverse transcribed using the High Capacity RNA-to-cDNA Kit (Applied Biosystems, Carlsbad, California, USA) according to the manufacturers’ instructions. For the epithelial cell samples, 500 ng of amplified RNA resulting from two rounds of amplification of the total extracted RNA was reverse transcribed. The cDNA was stored at −20°C. Expression levels of each gene were determined using manufacturer-optimized primer/probe mixes, PrimeTime qPCR Assays (20x; Integrated DNA Technologies (IDT), Coralville, Iowa, USA) and TaqMan Fast Advanced Master Mix (2x; Applied Biosystems) on the RotorGene 6000 qPCR instrument (Qiagen). Expression levels of the target genes were assessed relative to the Calnexin (*Canx*; NM_007597) reference gene, which had similar expression levels across all treatments as determined by microarray in this and other studies in the *Il10^−/−^* mouse model [33], [34].

All qPCR analyses (no-template controls and samples) were prepared as duplicate 10 µl reactions comprising a 9.0 µl aliquot of master mix (5.0 µl of TaqMan Fast Advanced Master Mix, 0.5 µl of PrimeTime qPCR assay for the gene of interest, 3.5 µl of nuclease-free water), and 1 µl of cDNA diluted 1 in 10 in nuclease-free water. The thermal profile used was 50°C for 2 minutes (UNG incubation), 95°C for 20 seconds (polymerase activation) and 40 cycles of [95°C for 3 seconds (denaturation) and 60°C for 30 seconds (anneal/extend)]. Each run was performed twice. The data were normalized to the reference gene and analysed for expression level changes using the Relative Expression Software Tool (REST) software version 2.0.13 [35]. Data are presented as relative FC (equivalent to the FC values presented for the microarray results), 95% confidence interval and P values for each gene and treatment comparison. Data were considered significant when absolute FC was greater than 1.5 and probability was less than 0.05.

## Results

### Bodyweight, Feed intake, and Body Condition

All mice sampled at 6 weeks of age had a GHS of 1 (healthy) at the beginning and end of the experiment. At inoculation (the beginning of the study), the body weight of the C57BL/6J mice was lower than that of the *Il10^−/−^* mice (means of 16.5 *vs.* 18.6 g respectively, P = 0.03; Table 1). After two days, on the day prior to euthanasia (the end of the experiment for this group of mice), the mean weight of the C57BL/6J mice was still lower than that of the *Il10^−/−^* mice (means of 17.7 *vs.* 19.6 g respectively, P = 0.02; Table 1). Only one intake measurement was made for this group of mice.

**Table 1:** Body weight and food intake data at the beginning and end of the experiment.

Parameter	Mean initial*	SD	P values for between-strain differences	Mean final#	SD	P values for between-strain differences
***Body weight (g)***						
6 week old C57BL/6J mice	16.5	2.0		17.7	1.7	
6 week old *Il10^−/−^* mice	18.6	1.6	0.03	19.6	1.4	0.02
12 week old C57BL/6J mice	17.0	1.6		23.7	1.9	
12 week old *Il10^−/−^* mice	18.8	1.8	0.08	24.2	1.3	0.50
***Food intake (g/day)***						
6 week old C57BL/6J mice	3.4	0.6		ND	ND	ND
6 week old *Il10^−/−^* mice	3.1	0.4	0.27	ND	ND	ND
12 week old C57BL/6J mice	3.7	0.7		3.0	0.2	
12 week old *Il10^−/−^* mice	3.0	0.6	0.04	3.1	0.3	0.40

* Initial weight measurements were made at inoculation. Initial intake was measured two days after inoculation.

# Final weight measurements were made on the day prior to sampling.

SD: standard deviation.

ND: no data.

The mice sampled at 12 weeks also had a GHS of 1 (healthy) at the beginning and end of the experiment. The *Il10^−/−^* mice developed minor changes in the fur condition on the back of their neck, with it becoming thinner and duller towards the end of the experiment. However, no other external changes or differences between strains were observed. There were no differences in the body weights of C57BL/6J and *Il10^−/−^* mice sampled at 12 weeks of age either at the beginning (P = 0.08) or end of the experiment (P = 0.50) (Table 1). Mean food intake was higher for the C57BL/6J mice at the beginning of the experiment (P = 0.04), but similar for both strains at the end of the experiment (P = 0.40).

### Histological Injury Score

Duodenum histology scores were low and showed no correlation between treatments, while jejunum and ileum scores were zero for all mice (data not shown). Colon histology and intestinal area data for each sampling time and mouse strain are shown in Table 2. Colon HIS was not different between *Il10^−/−^* and control mice at 6 weeks of age (0.17 *vs.* 0.28; P>0.05), but was significantly higher for *Il10^−/−^* mice compared to C57BL/6J mice (3.00 *vs.* 0.28, P<0.05) at 12 weeks. No mice of either strain at 6 weeks of age and/or any C57BL/6J mice at 12 weeks of age had any visible signs of inflammation throughout the length of their intestinal tract. All *Il10^−/−^* mice at 12 weeks had some intestinal thickening, indicating the presence of inflammation throughout the intestine. Intestinal area, with and without taking into account mouse weight at sampling, was larger for *Il10^−/−^* mice than C57BL/6J mice at 12 weeks of age (Table 2).

**Table 2:** Intestinal inflammation data by treatment.

Strain	Age (weeks)	Number ofmice	Mean colon histological injury score	Number of samples available for intestinal area#	Mean intestinalarea (mm^2^)	Mean intestinal area per gram body weight(mm^2^/g)
*Il10^−/−^*	6	9	0.17	3	1534	83.5
	12	10	3.00 *	9	1923*	86.1 *
C57BL/6J	6	8	0.28	3	1502	95.7
	12	8	0.28	8	1399	61.9

Mean colon histological injury score and mean intestinal area for each strain at each time point, showing signs of inflammation for the *Il10^−/−^* mice at 12 weeks of age.

* denotes a mean that is significantly (P<0.05) different for *Il10^−/−^* mice compared to C57BL/6J mice at 12 weeks of age. Predicted means for colon histological injury score were obtained from unbalanced ANOVA analysis performed on (log+0.05)-transformed data in GenStat v11, then predicted means were back-transformed to give the data presented here.

# Due to sampling time pressure, data was obtained for most mice at the 12-week sampling time, but only three for each strain at the 6-week sampling time. Observations of the appearance of the intestine were also recorded at sampling as another indicator of the degree and location of intestinal inflammation in each mouse.

### Colon Gene Expression by Microarray Analysis

#### Differentially expressed genes

In intact colon, the highest number of differentially expressed genes (3493; FC >1.5; FDR <0.05) as well as the highest fold changes, occurred in 12 week old *Il10^−/−^* mice compared to 12 week old C57BL/6J mice. A similar number of genes, 2309, were differentially expressed in 12 week old *Il10^−/−^* mice compared to 6 week old *Il10^−/−^* mice. In contrast, a lower number of genes were differentially expressed in C57BL/6J mice at 12 weeks compared to those at 6 weeks (251), and in 6 week old *Il10^−/−^* mice compared to 6 week old C57BL/6J mice (65). Fewer genes were differentially expressed for each comparison in the epithelial cell microarrays than the intact colon microarrays. The highest numbers of differentially expressed genes in epithelial cells also occurred in the 12 week old *Il10^−/−^* mice compared to 12 week old C57BL/6J mice (888) and 12 week old *Il10^−/−^* mice compared to 6 week old *Il10^−/−^* mice (385). Only 16 genes were differentially expressed in epithelial cells in 6 week old *Il10^−/−^* mice compared to 6 week old C57BL/6J mice. qPCR results confirmed the microarray FC and FDR/P values for the six genes measured (Table 3).

**Table 3:** qPCR validation of microarray results.

		*Il10^−/−^* mice, 12 weeks *vs*. *Il10^−/−^* mice, 6 weeks					*Il10^−/−^* mice, 12 weeks *vs*. C57BL/6J mice, 12 weeks			
	Gene	qPCR FC	qPCR P value	Microarray FC	Microarray FDR	Gene	qPCR FC	qPCR P value	Microarray FC	Microarray FDR
**Intact colon**	***Ppara***	−2.6	<0.001	−3.0	<0.001	***Ppara***	−2.6	<0.001	−2.4	0.002
	***Stat1***	2.0	<0.001	2.1	0.033	***Stat1***	3.1	<0.001	3.6	<0.001
	***S100g***	−62.5	<0.001	−99.2	<0.001	***S100g***	−15.6	<0.001	−21.0	<0.001
	***Il18***	−1.3	0.009*	−1.4	0.361	***Il18***	1.1	0.113	1.1	0.764
	***Mmp3***	6.6	<0.001	10.7	<0.001	***Mmp3***	5.3	<0.001	13.6	<0.001
	***Tap2***	1.9	<0.001	1.6	0.029	***Tap2***	2.2	<0.001	2.4	<0.001
**Colon epithelium**	***Stat1***	2.9	<0.001*	1.6	0.785	***Stat1***	6.3	<0.001	4.2	<0.001
	***S100g***	−6.5	<0.001*	−1.2	0.997	***S100g***	−4.3	0.032	−10.9	0.001
	***Il18***	−2.0	0.107	2.0	0.456	***Il18***	1.1	0.982	−1.4	0.768
	***Tap2***	1.6	0.488	−1.2	0.997	***Tap2***	−1.1	0.953	1.2	0.988

Table shows fold changes (FC) and P values (for qPCR data) or false discovery rates (FDR; for microarray data) for the six genes validated using qPCR for both intact colon and colon epithelium. All genes that were differentially expressed in the microarrays were differentially expressed according to qPCR and all fold changes were in the same direction in both qPCR and microarray.

* denotes genes that were significantly differentially expressed according to qPCR but not microarray.

**Figure 3:**
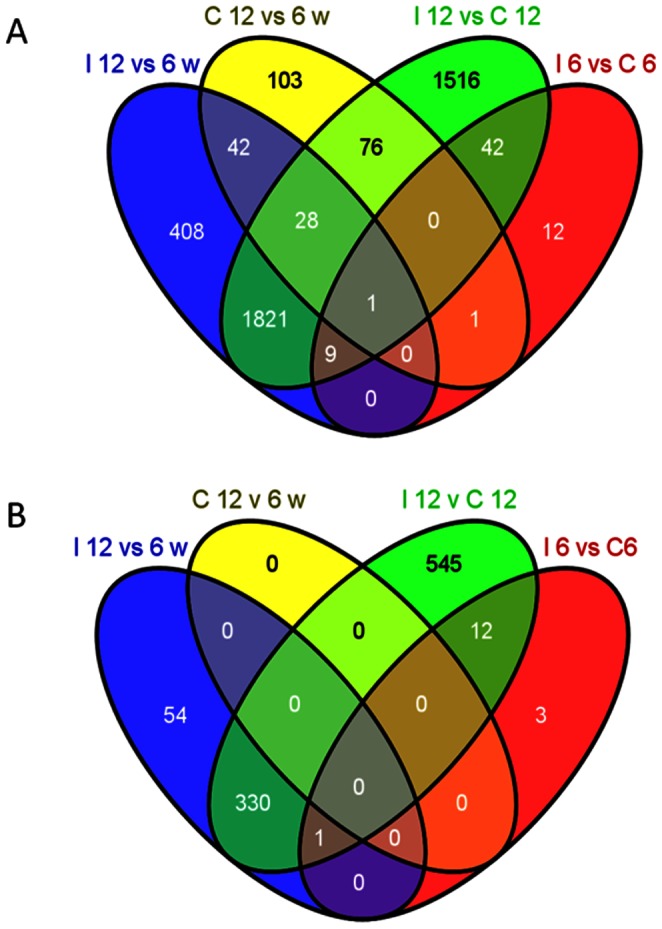
Overlap of differentially expressed genes between treatments. Venn diagrams show the numbers of differentially expressed genes in each comparison and how many are shared between each comparison, for intact colon (A) and colon epithelium (B). Treatments are identified in the diagram as: *Il10^−/−^* mice =  I; C57BL/6J mice =  C; weeks of age =  w. Each colour arbitrarily represents a different treatment. Numbers are of differentially expressed genes within each treatment comparison and each microarray experiment (intact colon and epithelial cells). Numbers in (B) are not subsets of the equivalent treatment comparison in (A), although there is overlap in the gene lists for each comparison between intact colon and colon epithelium.

The distribution of differentially expressed genes between groups for both intact colon and colon epithelium are shown in the Venn diagram in Figure 3. In the intact colon, the vast majority of differentially expressed genes occurred in the comparison between 12 week old *Il10^−/−^* mice and 12 week old C57BL/6J mice and approximately half of these genes were shared with the comparison of 12 week *vs.* 6 week old *Il10^−/−^* mice. Approximately 400 and 100 genes were unique to the 12 *vs.* 6 week comparisons for *Il10^−/−^* mice and C57BL/6J mice, respectively. In the colon epithelium, over half of the genes differentially expressed in the 12 week old *Il10^−/−^* mice *vs.*12 week old C57BL/6J mice were shared with the comparison of 12 week *vs.* 6 week old *Il10^−/−^* mice.

**Figure 4:**
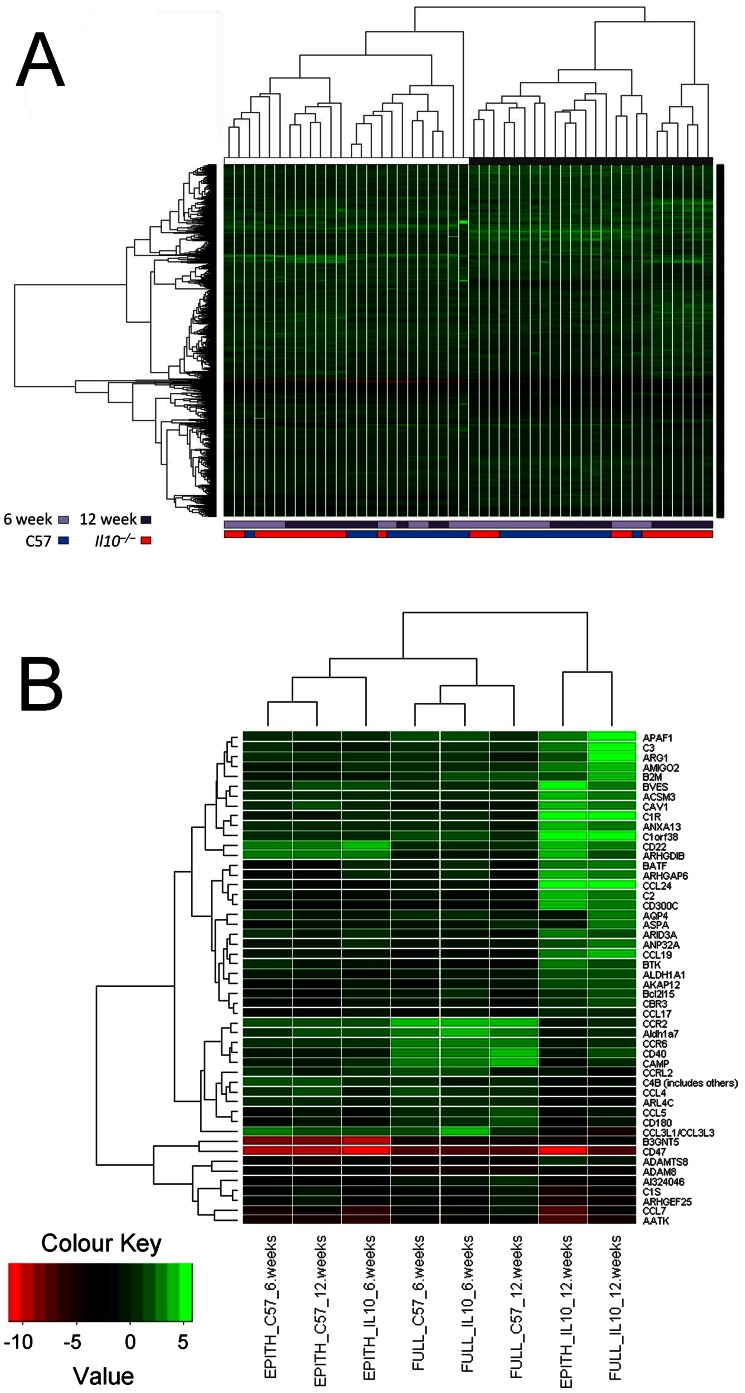
Overall and immune-related gene expression profiles in intact colon and epithelium. Heatmaps generated by unsupervised hierarchical clustering of differentially expressed genes. (A) shows all genes differentially expressed within any of the treatment comparisons, and the expression values are derived from individual mice. The bars above the heatmap indicate whether the data are from microdissected (white bar) or intact (black bar) tissue, while those below the heatmap show the age and genotype of the mice as indicated by the colour key. (B) shows a subset of differentially expressed immune related genes from the *Il10^−/−^* mice at 12 weeks *vs.* C57BL/6J mice at 12 weeks comparison, showing the gene expression profile of each treatment comparison in both intact colon and colon epithelium. Expression values for each gene are averages from 6 arrays per treatment.

Unsupervised hierarchical clustering was applied to two gene lists to generate heatmaps. For the first of these, all differentially expressed genes for any of the between-treatment comparisons were included, and data from individual mice was compared (Figure 4a). This identified two main groups, corresponding to either intact colon or to LMD samples. Within both of these groups, the *Il10^−/−^* mice at 12 weeks of age clustered together. *Il10^−/−^* mice at 6 weeks of age also clustered in the epithelium samples (LMD), whereas this was not seen in intact colon.

For the second comparison, a list of immune-related differentially expressed genes was plotted to visually show the clustering of treatments by similarity in gene expression profile (Figure 4b). Immune-related genes accounted for many of the differentially expressed genes for the *Il10^−/−^* mice at 12 weeks of age *vs.* C57BL/6J mice at 12 weeks of age and the *Il10^−/−^* mice at 12 weeks of age *vs. Il10^−/−^* mice at 6 weeks of age comparisons in both intact colon and colon epithelium. The gene expression profiles of *Il10^−/−^* mice at 12 weeks of age for intact colon and colon epithelium were similar to each other (Pearson correlation coefficient = 0.89), while the remaining profiles clustered by tissue type. In the other mice, tissue type (intact colon versus colon epithelium) had a greater contribution to the differentiation between expression profiles. All C57BL/6J mice and 6-week-old *Il10^−/−^* mice had similar profiles within intact colon and colon epithelium.

In the intact colon gene expression profiles, the 6-week-old C57BL/6J and *Il10^−/−^* mice were more similar to each other (Pearson correlation coefficient = 0.97) than to 12-week-old C57BL/6J mice (Pearson correlation coefficients = 0.96 and 0.91, respectively). In the epithelium, 6-week-old C57BL/6J mice were more similar to 12-week-old C57BL/6J mice (Pearson correlation coefficient = 0.97) than the 6-week-old *Il10^−/−^* mice (Pearson correlation coefficient = 0.94). This shows that in the epithelium, there is a greater difference evident in gene expression profiles in this subset of immune-related genes between *Il10^−/−^* mice and C57BL/6J mice at 6 weeks of age.

#### Pathway analysis

The top five canonical pathways (identified using IPA) for each comparison for intact colon and colon epithelium are presented in Table 4. The only comparisons for which significant pathways were identified were *Il10^−/−^* mice at 12 weeks of age versus C57BL/6J mice at 12 weeks of age and *Il10^−/−^* mice at 12 weeks of age versus C57BL/6J mice at 6 weeks of age. The percentages of genes in a pathway that were differentially expressed in any treatment comparison were smaller for the colon epithelium data (10–22%) than the intact colon (25–52%). There was overlap within the top 5 pathways in each treatment comparison for intact colon and colon epithelium. In the intact colon, all of the top 5 pathways were immune-related. In the epithelium, one of the top 5 pathways for each treatment comparison was not related to immune functioning (fatty acid metabolism).

**Table 4:** Top five canonical pathways for intact colon and colon epithelium.

Part of colon	Canonical pathway	Percentage of genes in pathwaythat were observed in thisgene list	P value
*Il10^−/−^ mice, 12 vs. 6 weeks*			
Intact colon	Communication between innate and adaptive immune cells	36	9.54×10^−24^
	Altered T and B cell signalling in rheumatoid arthritis	42	1.40×10^−20^
	Graft-versus-host disease signalling	50	2.73×10^−17^
	T helper cell differentiation	44	1.62×10^−16^
	Dendritic cell maturation	25	3.68×10^−15^
Colon epithelium	Dendritic cell maturation	10	1.95×10^−11^
	T helper cell differentiation	18	1.06×10^−10^
	Altered T and B cell signalling in rheumatoid arthritis	15	1.28×10^−10^
	Graft-vs-host disease signalling	22	2.11×10^−10^
	Type I diabetes mellitus signalling	12	3.64×10^−09^
*Il10^−/−^ mice vs. C57BL/6J mice,12 weeks*			
Intact colon	Communication between innate and adaptive immune cells	38	7.42×10^−19^
	Dendritic cell maturation	31	4.49×10^−16^
	Altered T and B cell signalling in rheumatoid arthritis	45	1.19×10^−15^
	Allograft rejection signalling	29	5.82×10^−14^
	Graft-vs-host disease signalling	52	6.70×10^−14^
Colon epithelium	LPS/IL-1 mediated inhibition of RXR function	14	2.92×10^−12^
	Fatty acid metabolism	13	5.78×10^−12^
	Allograft rejection signalling	16	4.12×10^−11^
	Communication between innate and adaptive immune cells	16	4.23×10^−10^
	Dendritic cell maturation	13	6.00×10^−10^

Canonical pathways were generated by IPA from the genelists for each treatment comparison. The comparisons were: *Il10^−/−^* mice at 12 weeks of age *vs.* C57BL/6J mice at 12 weeks of age; *Il10^−/−^* mice at 12 weeks of age *vs. Il10^−/−^* mice at 6 weeks of age; C57BL/6J mice at 12 weeks of age *vs.* C57BL/6J mice at 6 weeks of age; and *Il10^−/−^* mice at 6 weeks of age *vs.* C57BL/6J mice at 6 weeks of age.

**Figure 5:**
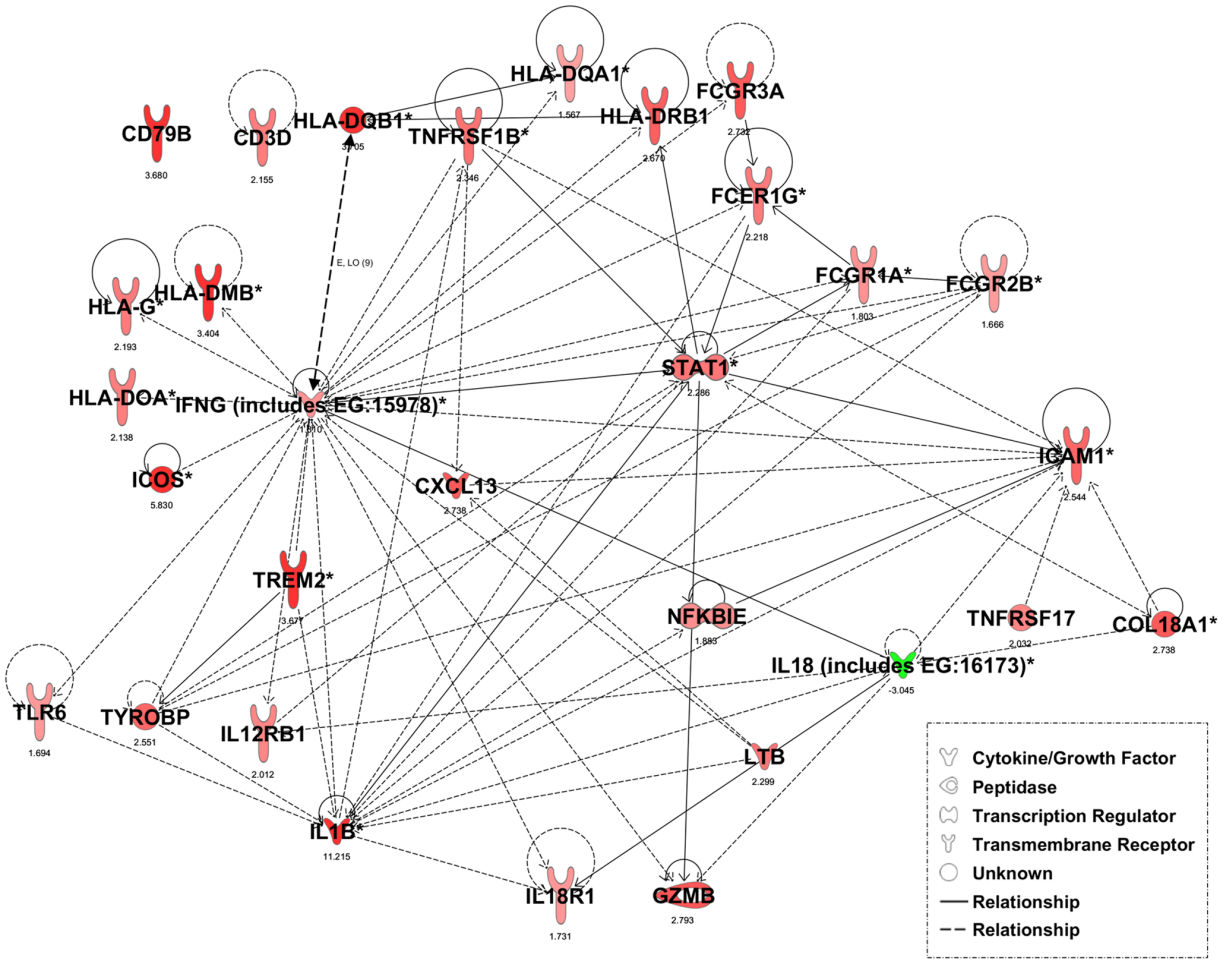
Network diagram showing gene expression in the colon epithelium. Network diagram of genes from the top 5 differentially expressed canonical pathways in *Il10^−/−^* mice at 12 weeks of age *vs.* 6 weeks of age in the colon epithelium. *denotes genes that are detected two or more times on an array. Genes or gene products are represented as nodes, and the biological relationship between two nodes is represented as a line. All relationships are supported by at least one reference from literature. Red and green coloured nodes indicate degree of fold change, with red indicating an increase in expression in *Il10^−/−^* mice at 12 weeks of age relative to *Il10^−/−^* mice at 6 weeks of age and green indicating a decrease in expression. Colour intensity is correlated with the degree of change in expression with greater intensity representing a higher expression level. Nodes are displayed with various shapes that represent the functional class of genes as shown in the legend. Fold changes are shown beneath each node.

**Figure 6:**
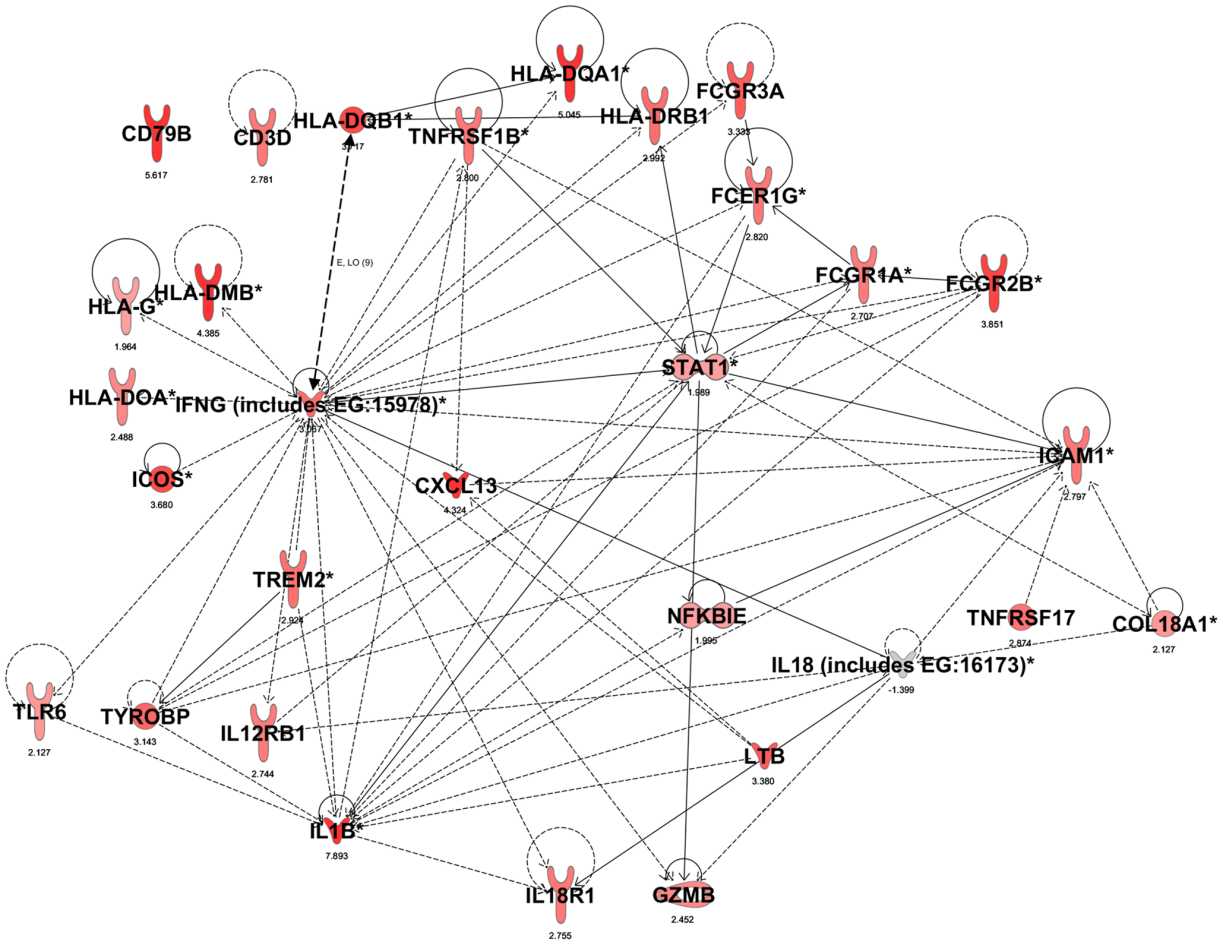
Network diagram of gene expression in the intact colon. Network diagram shown in Figure 5 overlaid with expression values for the same treatment comparison in intact colon.

A network of the molecules from the top 5 differentially expressed canonical pathways in the colon epithelium for *Il10^−/−^* mice at 12 weeks of age compared to 6 weeks of age is shown in Figure 5. The same network overlaid with expression values for the same comparison in intact colon is shown in Figure 6 and shows that these genes had similar patterns of expression in both intact colon and colon epithelium. One major difference was that the abundance of IL18 mRNA was reduced in the colon epithelium of *Il10^−/−^* mice at 12 weeks of age compared to 6 weeks of age but was not differentially expressed for the intact colon. These findings indicate that the gene expression profiles in *Il10^−/−^* mice at 12 weeks of age were mostly similar between intact colon and colon epithelium.

#### Gene set enrichment analysis

Differentially expressed genes clustered into few gene sets in *Il10^−/−^ vs.* C57BL/6J mice at 6 weeks of age: 3 in intact colon and 6 in colon epithelium (Table 5), with only one of these sets still significant after correction for multiple hypothesis testing. This was not surprising because there was no difference in colon HIS between these groups. In the colon epithelium, 2 of the 6 gene sets were immune-related (chemokine signalling pathway, and cytokine-cytokine receptor interaction) while in the intact colon, no immune-related gene sets were identified. This may indicate that targeting epithelial cells can increase sensitivity for detecting immune changes that occur early in the inflammatory process. Differentially expressed genes also clustered into few gene sets in C57BL/6J mice at 12 weeks of age *vs.* C57BL/6J mice at 6 weeks of age (Table 6), none of which were significant after correction for multiple hypothesis testing. These gene sets were related to cell membrane function and cell signalling and likely represented age and growth-related changes in the colon of the C57BL/6J mice.

**Table 5:** sets for *Il10^−/−^* mice *vs.* C57BL/6J mice at 6 weeks of age.

Pathway ID	Pathway name	P value	FDR
*Intact colon*			
mmu00520	Amino sugar and nucleotide sugar metabolism	0.016	1
mmu04141	Protein processing in endoplasmic reticulum	0.020	1
mmu04914	Progesterone-mediated oocyte maturation	0.031	1
*Colon epithelium*			
mmu00565	Ether lipid metabolism	<0.001	<0.001
mmu04062	Chemokine signalling pathway	0.004	0.644
mmu04914	Progesterone-mediated oocyte maturation	0.020	1
mmu05144	Malaria	0.033	1
mmu04110	Cell cycle	0.035	1
mmu04060	Cytokine-cytokine receptor interaction	0.048	1

Gene sets were obtained from GSEA for intact colon and colon epithelium.

**Table 6:** Gene sets for C57BL/6J mice at 12 weeks of age *vs*. 6 weeks of age.

Pathway ID	Pathway name	P value	FDR
*Intact colon*			
mmu04540	Gap junction	0.002	0.298
mmu04080	Neuroactive ligand-receptor interaction	0.005	0.801
mmu04670	Leukocyte transendothelial migration	0.020	1
*Colon epithelium*			
mmu00512	O-Glycan biosynthesis	0.012	1
mmu04070	Phosphatidylinositol signaling system	0.020	1
mmu00562	Inositol phosphate metabolism	0.039	1
mmu03010	Ribosome	0.040	1

Gene sets were obtained from GSEA for intact colon and colon epithelium.

In *Il10^−/−^ vs.* C57BL/6J mice at 12 weeks of age, and in *Il10^−/−^* mice at 12 weeks *vs.* 6 weeks, differentially expressed genes clustered into a large number of gene sets which was expected given the change in colon HIS. A number of these pathways were related to immunity and cancer. There were fewer metabolism-related gene sets in *Il10^−/−^* mice at 12 weeks *vs.* 6 weeks than *Il10^−/−^ vs.* C57BL/6J mice at 12 weeks of age. Overall, differentially expressed genes clustered into similar gene sets in the colon epithelium and intact colon for both comparisons of *Il10^−/−^* mice at 12 weeks *vs.* C57BL/6J mice at 12 weeks (Table 7) and *Il10^−/−^* mice at 12 weeks *vs. Il10^−/−^* mice at 6 weeks (Table 8).

**Table 7:** Gene sets for *Il10^−/−^* mice *vs*. C57BL/6J mice at 12 weeks of age.

KEGG pathway ID	Pathway name	P value	FDR
*Intact colon*			
mmu00051	Fructose and mannose metabolism	<0.001	<0.001
mmu00520	Amino sugar and nucleotide sugar metabolism	<0.001	<0.001
mmu00562	Inositol phosphate metabolism	<0.001	<0.001
mmu00565	Ether lipid metabolism	<0.001	<0.001
mmu00601	Glycosphingolipid biosynthesis - lacto and neolacto series	<0.001	<0.001
mmu04010	MAPK signaling pathway	<0.001	<0.001
mmu04012	ErbBsignaling pathway	<0.001	<0.001
mmu04060	Cytokine-cytokine receptor interaction	<0.001	<0.001
mmu04062	Chemokine signaling pathway	<0.001	<0.001
mmu04115	p53 signaling pathway	<0.001	<0.001
mmu04120	Ubiquitin mediated proteolysis	<0.001	<0.001
mmu04142	Lysosome	<0.001	<0.001
mmu04145	Phagosome	<0.001	<0.001
mmu04150	mTORsignaling pathway	<0.001	<0.001
mmu04210	Apoptosis	<0.001	<0.001
mmu04370	VEGF signaling pathway	<0.001	<0.001
mmu04512	ECM-receptor interaction	<0.001	<0.001
mmu04514	Cell adhesion molecules (CAMs)	<0.001	<0.001
mmu04612	Antigen processing and presentation	<0.001	<0.001
mmu04620	Toll-like receptor signaling pathway	<0.001	<0.001
*Colon epithelium*			
mmu00565	Ether lipid metabolism	<0.001	<0.001
mmu00601	Glycosphingolipid biosynthesis - lacto and neolacto series	<0.001	<0.001
mmu04010	MAPK signaling pathway	<0.001	<0.001
mmu04060	Cytokine-cytokine receptor interaction	<0.001	<0.001
mmu04062	Chemokine signaling pathway	<0.001	<0.001
mmu04145	Phagosome	<0.001	<0.001
mmu04210	Apoptosis	<0.001	<0.001
mmu04270	Vascular smooth muscle contraction	<0.001	<0.001
mmu04370	VEGF signaling pathway	<0.001	<0.001
mmu04514	Cell adhesion molecules (CAMs)	<0.001	<0.001
mmu04612	Antigen processing and presentation	<0.001	<0.001
mmu04620	Toll-like receptor signaling pathway	<0.001	<0.001
mmu04621	NOD-like receptor signaling pathway	<0.001	<0.001
mmu04623	Cytosolic DNA-sensing pathway	<0.001	<0.001
mmu04630	Jak-STAT signaling pathway	<0.001	<0.001
mmu04640	Hematopoietic cell lineage	<0.001	<0.001
mmu04650	Natural killer cell mediated cytotoxicity	<0.001	<0.001
mmu04660	T cell receptor signaling pathway	<0.001	<0.001
mmu04662	B cell receptor signaling pathway	<0.001	<0.001
mmu04664	Fc epsilon RI signaling pathway	<0.001	<0.001

Gene sets were obtained from GSEA for intact colon and colon epithelium. The top 20 gene sets are shown.

**Table 8:** Gene sets for *Il10^−/−^* mice at 12 weeks of age *vs.* 6 weeks of age.

KEGG pathway ID	Pathway name	P value	FDR
*Intact colon*			
mmu00601	Glycosphingolipid biosynthesis - lacto and neolacto series	<0.001	<0.001
mmu04012	ErbBsignaling pathway	<0.001	<0.001
mmu04060	Cytokine-cytokine receptor interaction	<0.001	<0.001
mmu04062	Chemokine signaling pathway	<0.001	<0.001
mmu04142	Lysosome	<0.001	<0.001
mmu04144	Endocytosis	<0.001	<0.001
mmu04145	Phagosome	<0.001	<0.001
mmu04210	Apoptosis	<0.001	<0.001
mmu04370	VEGF signaling pathway	<0.001	<0.001
mmu04514	Cell adhesion molecules (CAMs)	<0.001	<0.001
mmu04612	Antigen processing and presentation	<0.001	<0.001
mmu04620	Toll-like receptor signaling pathway	<0.001	<0.001
mmu04621	NOD-like receptor signaling pathway	<0.001	<0.001
mmu04630	Jak-STAT signaling pathway	<0.001	<0.001
mmu04640	Hematopoietic cell lineage	<0.001	<0.001
mmu04650	Natural killer cell mediated cytotoxicity	<0.001	<0.001
mmu04660	T cell receptor signaling pathway	<0.001	<0.001
mmu04662	B cell receptor signaling pathway	<0.001	<0.001
mmu04664	Fc epsilon RI signaling pathway	<0.001	<0.001
mmu04666	Fc gamma R-mediated phagocytosis	<0.001	<0.001
*Colon Epithelium*			
mmu00562	Inositol phosphate metabolism	<0.001	<0.001
mmu04060	Cytokine-cytokine receptor interaction	<0.001	<0.001
mmu04062	Chemokine signaling pathway	<0.001	<0.001
mmu04145	Phagosome	<0.001	<0.001
mmu04210	Apoptosis	<0.001	<0.001
mmu04514	Cell adhesion molecules (CAMs)	<0.001	<0.001
mmu04612	Antigen processing and presentation	<0.001	<0.001
mmu04620	Toll-like receptor signaling pathway	<0.001	<0.001
mmu04621	NOD-like receptor signaling pathway	<0.001	<0.001
mmu04640	Hematopoietic cell lineage	<0.001	<0.001
mmu04650	Natural killer cell mediated cytotoxicity	<0.001	<0.001
mmu04660	T cell receptor signaling pathway	<0.001	<0.001
mmu04662	B cell receptor signaling pathway	<0.001	<0.001
mmu04664	Fc epsilon RI signaling pathway	<0.001	<0.001
mmu04666	Fc gamma R-mediated phagocytosis	<0.001	<0.001
mmu04672	Intestinal immune network for IgA production	<0.001	<0.001
mmu04730	Long-term depression	<0.001	<0.001
mmu04940	Type I diabetes mellitus	<0.001	<0.001
mmu05140	Leishmaniasis	<0.001	<0.001
mmu05142	Chagas disease	<0.001	<0.001

Gene sets were obtained from GSEA for intact colon and colon epithelium. The top 20 gene sets are shown.

#### Expression of known IBD genes in intact colon and colon epithelium

From a list of 32 genes whose expression is known to be altered in the inflamed intestinal mucosa in IBD patients [36], [37], [38], [39], the majority were also differentially expressed in this experiment. These genes included many interleukins, chemokine receptor and ligands, metalloproteinases, and genes commonly identified in IBD and other inflammatory diseases (Table 9). The majority of these 32 genes were differentially expressed in the intact colon (91% for the comparison *Il10^−/−^* mice at 12 weeks of age *vs.* C57BL/6J mice at 12 weeks of age and 84% for the comparison *Il10^−/−^* mice at 12 weeks of age *vs. Il10^−/−^* mice at 6 weeks of age). This confirms that the pathology of the mouse model used here bears many similarities to IBD and to commonly used mouse models of IBD [7], [14]. In the colon epithelium, 69% of these genes were differentially expressed for *Il10^−/−^* mice at 12 weeks of age *vs.* C57BL/6J mice at 12 weeks of age, compared to 91% in intact colon, indicating that the gene expression profiles were similar, but not identical, in colon epithelium compared to intact colon. However, in *Il10^−/−^* mice at 12 weeks of age *vs. Il10^−/−^* mice at 6 weeks of age, only 34% of these genes were differentially expressed in the colon epithelium compared to 84% in intact colon, indicating there were differences in the gene expression profile between epithelium and intact colon in *Il10^−/−^* mice, from the early stage of colitis to when colitis was fully developed.

**Table 9:** Expression of IBD genes in colon and colon epithelium in *Il10^−/−^* mice.

	*Il10^−/−^ vs.C57BL/6J mice, 12 weeks*		*Il10^−/−^ mice, 12 vs.6 weeks*	
IBD-related genes	Intactcolon	Colonepithelium	Intactcolon	Colonepithelium
Tumour necrosis factor; *TNF*	X		X	
Interferon-γ; *IFNγ*	X	X	X	X
Lymphotoxin β; *Ltb*	X	X	X	X
Interleukin-6; *IL6*	X	X	X	
Interleukin-16; *IL16*			X	X
Interleukin-18 receptor 1; *IL18R1*	X		X	X
Interleukin-22; *IL22*	X			
Chemokine receptor 2; *CCR2*	X	X	X	X
Chemokine receptor 7; *CCR7*	X		X	
Chemokine (C-C motif) ligand 2; *CCL2*	X	X		X
Chemokine (C-C motif) ligand 3; *CCL3*	X	X	X	X
Chemokine (C-C motif) ligand 4; *CCL4*	X	X	X	
Chemokine (C-C motif) ligand 5; *CCL5*	X	X	X	X
Chemokine (C-C motif) ligand 7; *CCL7*	X		X	
Chemokine (C-C motif) ligand 11; *CCL11*				
Chemokine (C-C motif) ligand 17; *CCL17*	X	X	X	
Chemokine (C-C motif) ligand 20; *CCL20*			X	
Chemokine (C-X-C motif) receptor 3; *CXCR3*	X	X	X	
Chemokine (C-X-C motif) ligand 1; *CXCL1*	X	X	X	
Chemokine (C-X-C motif) ligand 5; *CXCL5*	X	X	X	
Chemokine (C-X-C motif) ligand 10; *CXCL10*	X	X		
Matrix metalloproteinase 3; *MMP3*	X	X	X	X
Matrix metalloproteinase 7; *MMP7*	X	X	X	
Matrix metalloproteinase 9; *MMP9*	X	X	X	
Matrix metalloproteinase 14; *MMP14*	X	X	X	
Tissue inhibitor of metalloproteinase 1; *TIMP1*	X	X	X	
Regenerating islet-derived 3γ; *REG3G*	X	X	X	
Pancreatitis-associated protein; *PAP*	X	X	X	
S-100 calcium binding protein A8: *S100A8*	X	X	X	X
S-100 calcium binding protein A9: *S100A9*	X	X	X	X
ATP-binding cassette, subfamily B, 1a; *Abcb1a*	X		X	
Prostaglandin-endoperoxide synthase 2; *PTGS2*	X		X	
TOTAL DIFFERENTIALLY EXPRESSED GENES (OUT OF 32)	29	22	27	11

Genes identified as having increased expression levels in colon tissue in CD and/or UC patients with indication of whether they were also differentially expressed in the *Il10^−/−^* mice at 12 week of age *vs.* C57BL/6J mice at 12 weeks or *vs. Il10^−/−^* mice at 6 weeks of age. An X denotes a gene that is differentially expressed while a blank space indicates that the gene was not significantly differentially expressed in the dataset.

## Discussion

Findings from this study indicate that microarray analysis of intact colon and colon epithelium in colon inflammation in the *Il10^−/−^* mouse model produces similar gene expression profiles at the pathway and gene set level. Overall, intact colon samples provided information on a greater number of genes, but in early inflammation, microdissected epithelium profiles were more sensitive to changes in immune-related pathways.

Colon histology results confirmed that the *Il10^−/−^* mice used in this experiment developed a phenotype of colon inflammation, similar to that reported for this mouse model [14], [16], [33]. In intact colon, the gene expression patterns in 12 week old *Il10^−/−^* mice compared to age-matched C57BL/6J mice, or 6 week old *Il10^−/−^* mice, were consistent with other studies of colon gene expression in the *Il10^−/−^* model [14], [34], [40] and in other mouse models of IBD [7]. These results confirm that these mice are a relevant model in which to study the gene expression profile of intact colon and the colon epithelium during colitis.

### Many IBD Genes were Differentially Expressed in the Colon Epithelium as well as Intact Colon

Genes whose expression level is known to be increased in IBD were increased in the colon epithelium of *Il10^−/−^* mice at 12 weeks of age, including: cytokine and cytokine receptor genes (*Ifn*-γ, *Ltβ, Il6, Il16, Il18r1*), and chemokine and chemokine receptor genes (chemokine (C-C motif) receptor (*Ccr*)-*2,* chemokine (C-C motif) ligand (*Ccl*)-2, *3, 4, 5, 17,* chemokine (C-X-C motif) receptor (*Cxcr*)-3, and chemokine (C-X-C motif) ligand (*Cxcl*)-*1, 5, 10*). Chemokines control leukocyte trafficking and the migration of leukocytes into sites of inflammation are crucial for the pathogenesis of experimental colitis and increased gene and protein abundance of chemokines occurs in IBD [41], [42]. The results from this study are in agreement with reports that the colon epithelium produces chemokine signals to induce an influx of leukocytes in mucosal inflammation [43].

Genes involved in tissue remodelling (matrix metalloproteinase (*Mmp*)-*3, 7, 9, 14,* and tissue inhibitor of metalloproteinase-1 (*Timp1*)) were also expressed in the epithelium in inflamed *Il10^−/−^* mice at 12 weeks of age. Matrix metalloproteinases (MMPs) are involved with tissue remodelling, angiogenesis, and promotion of leukocyte extravasation and their expression is increased in IBD [44], [45]. The activity of MMP1, 3 and 9 is controlled by tissue inhibitor of metalloproteinase (TIMP-1), the expression of which is also increased in colitis [45]. Increased production of MMPs has a role in tissue damage in IBD and some studies show that genetic variation in MMP genes may play a role in inter-individual differences in UC susceptibility and clinical outcome [46]. The results of this study indicate that increased expression levels of gene transcripts for MMP and TIMP1 proteins in IBD occurs in the epithelium.

Other genes whose expression levels are known to be increased in IBD that were increased in the *Il10^−/−^* mice at 12 weeks of age in colon epithelium were the S-100 calcium binding proteins A8 and A9 (calgranulin A and B) (*S100a8, 9). S100a8* and *S100a9* form a heterodimer called calprotectin, a pro-inflammatory mediator involved in acute and chronic inflammation [47], [48]. This finding is in agreement with studies that showed that *S100a8* and *S100a9* are expressed in mucosal epithelium under inflammatory conditions and in inflammation-associated cancer [47], [48]. Increased expression levels of these genes in colitis may reflect neutrophil infiltration [7]. These transcripts could be localised to specific cell types within the epithelium, such as intraepithelial lymphocytes, or to neutrophils infiltrating the lamina propria which may have been inadvertently captured during microdissection, by comparing their relative expression levels in each cell type in a future study using LMD.

### Some IBD Genes were Differentially Expressed in Intact Colon but not Epithelium

Approximately 45% of the differentially expressed genes in the colon epithelium were also differentially expressed in intact colon for the comparisons between *Il10^−/−^* mice at 12 weeks *vs. Il10^−/−^* mice at 6 weeks, and *Il10^−/−^* mice at 12 weeks *vs.* C57BL/6J mice at 12 weeks. This suggests that either the similarity in pathways, processes and gene sets between intact colon and colon epithelium in these comparisons is due to fewer than 50% of the differentially expressed genes in the colon epithelium, or that many of the total number of differentially expressed genes in the epithelium belong to similar immune-related pathways. In the *Il10^−/−^* mice at 6 weeks *vs.* C57BL/6J mice at 6 weeks, 27% of differentially expressed genes in the colon epithelium were also differentially expressed in intact colon.

From the list of IBD-related genes, those that were not differentially expressed in the colon epithelium were: *Tnf*, *Il6*, *Il22*, *Ccl*-4, 7, 11, 17, and 20, *Cxcr3*, *Cxcl*-1, 5 and 10; *Mmp*-7, 9, and 14, *Timp1*, regenerating islet-derived 3γ (*Reg3γ*), pancreatitis-associated protein (*Pap*), ATP-binding cassette, subfamily B (MDR/TAP), 1a (*Abcb1a*), and prostaglandin-endoperoxide synthase 2 (*Ptgs2*). The lack of differential expression of *Abcb1a* in the epithelium was surprising. This gene codes for a membrane-associated transporter protein whose expression is altered in UC [49] and mouse models of colitis [7], and was differentially expressed in intact colon. Therefore, it was expected that this gene would be differentially expressed in the epithelium in the *Il10^−/−^* mice at 12 weeks of age compared to C57BL/6J mice or *Il10^−/−^* mice at 6 weeks of age. In microarray experiments performed with RNA from low numbers of cells, there can be increased variability in the expression ratios of genes and an increased likelihood of transcription error, which could result in inaccurate results for some mRNAs [50]. This could have occurred for *Abcb1a* and other IBD-associated genes in the epithelium, and if so, would be an argument for utilising intact colon tissue for IBD gene expression profiling in mouse models in order to obtain greater RNA yields.

### Intact Colon Samples Provide a Good Representation of Mucosal Gene Expression Changes

Overall, genes that have been identified in other studies as being differentially expressed in IBD were well-represented in these microarray experiments for both intact colon and colon epithelium. This indicates that intact colon samples provide a good indication of changes in gene expression profile that are occurring in the mucosa, in terms of gene sets and pathways. In the epithelium, when genes were not differentially expressed in a particular treatment comparison, closely related genes often were differentially expressed. For example, in colon epithelium for *Il10^−/−^* mice at 12 weeks of age *vs.* C57BL/6J mice at 12 weeks of age, *Mmps7, 9* or *14* were not differentially expressed but *Mmp10* was. Also, while *Tnf* itself was not differentially expressed, TNF receptor genes were. This indicates that differentially expressed genes in both intact colon and epithelium are involved in the same pathways and processes.

### Cellular Origin of Gene Expression changes in Colon Mucosa

The epithelial cell data was more sensitive to immune changes between mouse strains at 6 weeks of age using the gene set enrichment analysis. An example is ether lipid metabolism, which was significant in the *Il10^−/−^* vs. C57 comparison at 6 weeks only in the epithelium, whereas for the *Il10^−/−^* vs. C57 comparison at 12 weeks it was significant in both intact colon and epithelium. Some of the ether lipids (plasmalogens) might have a role in defence against oxidative damage [51], [52], and this may represent a very early stage of the inflammatory process. However, the colon epithelium had fewer differentially expressed genes from the group of 32 known IBD-related genes than did intact colon, particularly in the treatment comparison *Il10^−/−^* mice at 12 weeks of age *vs. Il10^−/−^* mice at 6 weeks of age. None of the listed IBD-related genes were differentially expressed at 6 weeks of age between *Il10^−/−^* and C57BL/6J mice in the colon epithelium, so increased mRNA abundance at 6 weeks of age was unlikely to be the reason they were not differentially expressed between 12 and 6 weeks of age in *Il10^−/−^* mice. This may indicate that they were more strongly expressed in non-epithelial cell types in colitis. When designing an experiment to analyse gene expression changes in *Il10^−/−^* mice in the early stages of colitis, it may be worth considering analysing the colon epithelium instead of intact colon in order to focus on early epithelial changes, with the understanding that information about some IBD-related genes may be missed.

The mRNA transcripts from the immune genes that were differentially expressed in the colon epithelium between inflamed *Il10^−/−^* mice and the other non-inflamed mice were likely to come from the enterocytes which form the majority of the collected cell population, or from intraepithelial lymphocytes (IEL). Increased pro-inflammatory cytokine secretory activity of IELs during colitis could account for the increased immune-related gene expression levels in epithelia from inflamed mouse colon. Intestinal IELs are important for the maintenance of intestinal homeostasis [53], [54], [55] and release cytokines upon activation that contribute to the activation and the recruitment of innate immune cells [56], [57]. The relative contribution of the IELs to the gene expression profile of the colon epithelium in *Il10^−/−^* mice is unknown. It is likely, based on the higher percentage of IL23R^+^ CD8^+^ IEL observed in CD and UC patients compared to healthy controls [57], that some types of IEL were more abundant in the inflamed colon.

The similarity between intact colon and colon epithelium gene expression profiles in *Il10^−/−^* mice *vs.* control mice in late inflammation in this study suggests that the colon epithelium (including IELs) is important in inflammatory signalling in IBD. Contamination of the microdissected epithelial cells by immune cells from the lamina propria was possible, and could provide an alternative explanation for the presence of differentially expressed immune-related genes and pathways in the epithelial cell microarrays. Future gene expression profiling of the colon epithelium should include a comparison of microdissected lamina propria gene expression profiles for genes known to be predominantly expressed in each compartment to confirm the specificity of the cell fractions, although this would not completely rule out some degree of cross-contamination [58]. In addition, the relative contribution of the epithelium compared to the immune cell infiltrates could be studied by comparing the gene expression of epithelial cells with immune cell aggregates microdissected from the same inflamed colon sections.

### Conclusions

Results from microarray analysis of intact colon in this experiment are consistent with other data in this and similar models indicating the suitability of the samples collected here for studying epithelial cell function in colitis. The findings from this study indicate that when analysed at the pathway level, intact colon is an appropriate tissue in which to examine gene expression changes in the mucosa in fully-developed colitis, as it produces similar gene expression profiles to the epithelium alone and captures information about a greater number of IBD-associated genes. Findings from this study also indicate that studying epithelial cell gene expression profiles, as opposed to intact colon profiles, may be more relevant when studying earlier stages of inflammation because isolation of specific cells may improve the ability to detect early changes in cell function (at the pathway level) that may lead to the development of inflammation.

Finally, while LMD shows promise as a tool to better understand IBD via *in vivo* models, it may be of relatively limited clinical use in terms of early diagnosis of IBD. This is because it relies on obtaining tissue samples (for example from a biopsy), which represents an invasive approach that is unlikely to be used for early diagnosis. However, it does demonstrate the importance of working with a well-defined subset of cells to identify early changes that are indicative of an inflammatory response, and this could potentially be applied to epithelial cells collected from fecal samples, or peripheral blood mononuclear cells [59]. This may enable early detection of inflammation in a relatively non-invasive manner for those who are at higher risk of developing IBD.
